# Cysts

**Published:** 1887-04

**Authors:** 


### CYSTS.

TRANSLATIONS BY A. McNEIL, M. D., SAN FRANCISCO, CAL.

(The following cases I translate from the *Allgemeine Hom. Zeitung* for the year 1867. They are reported by X. Bourgeos, M. D., and were published in *L'Art Médicale*, and translated into the German.)

### CYST OF THE KNEE.

May 26th, 1857, I was called to see a nun. She was forty-one; chloro-ænemic, and subject to frequent attacks of neuralgia and oppression. Menses profuse. Leucorrhœa.

For half a year she has had a constantly increasing swelling on the anterior aspect of the right knee. It is the size of a hen's egg, is movable, circumscribed, tense, and fluctuates easily. The skin covering it is dark red, and penetrated by bluish veins. Some pain on pressure; walking not affected, but to kneel on that knee is difficult.

Calc. carb.$^{30}$, twelve pellets in one hundred and twenty grammes of water, daily two spoonfuls.

I saw her in fourteen days. The first few days stitches in the tumor. It had become tenser and warmer. I discovered a decrease of the swelling, and less tension objectively. Fluctuation clearer. The skin covering the hygroma revealed folds.

Calc. carb.$^{200}$, twelve pellets, as before.

In three weeks more the tumor had completely disappeared. The skin covering the knee is soft; not the least trace of fluid.

The cure is perfect, no return. At the same time the menses have so far improved that they are no longer too early nor too profuse.

Julia P., thirty-six years old, has excellent health; consulted me on the 10th of September, 1860, on account of a hygroma of the right knee. A year before she had a bruise thereon from a blow, which had been treated by compresses wet with spirits of wine and camphor. Some swelling had remained, and this by degrees formed the above-mentioned tumor. It is as large as the third of a hen's egg. It is circumscribed, painless, fluctuating. The skin of the tumor is not discolored. Walking is more difficult. She tires easily.

Calc. carb.$^{30}$, four drops in one hundred and twenty grammes of water, two tablespoonfuls a day.

September 29th, nineteen days after the first visit, the cyst is smaller, softer, with a tendency to retrogression. Sac. lac.

In fourteen days more the cyst has almost disappeared, the the skin thereon is relaxed, and lying in folds. No fluctuation; it appeared as if the walls were in contact, and glided over each other.

In November the entire disappearance of the tumor was ascertained.

### Cyst on the Finger.

Pastor C., fifty-five years old, good constitution, has suffered a long time from gastralgia, and for two years has had a small swelling on the phalangeal joint of the middle finger of the right hand. The tumor is soft, movable, fluctuating, and of the size of a hazel-nut. It is not painful, but hinders the movement of the fingers. For some months it has increased. One physician had advised that the serous sac be removed by an operation.

I preferred internal medication. January 18th, 1864, I ordered three pellets Calc. carb.$^{30}$, night and morning, dissolved in a spoonful of water, for six days.

Two months after the pastor assured me that it was completely cured, the cyst having disappeared in ten days. Only the loose skin indicates the presence of the former ganglion.

Such chronic diseases serve better than acute diseases as evidence of the efficiency of the infinitesimal dose. Will any one speak of a cure by nature in the case of a cyst which has lasted two years and constantly growing, when it disappears in fourteen days after the administration of a remedy? Is it not much more the evident effect of a positive medicinal action?

### Cyst on the Wrist.

G., otherwise of good health, suffers from time to time from gastralgia, constipation, and dyspepsia. He has been repeatedly attacked by pemphigus. The bullæ burst, and left painful ulceration, which continued several weeks. Under Mercur. and Arsen. in infinitesimal doses the disease disappeared, but relapses occurred, which, however, disappeared sooner each time.

He had a cyst the size of a hazel-nut on the anterior aspect of the wrist, which he had punctured every three months. I had done it twice myself. I proposed internal treatment, to which he acceded, in order that he might see the effect of homœopathic remedies.

July 10th, 1865, ten days after puncture I gave Calc. carb.$^{30}$ and after three weeks the same.

In four months I saw it again. He showed me his wrist, and there was not the slightest trace of the ganglion. I afterward convinced myself that there had been no relapse.

### Cyst on the Eyelid.

Emil B., twenty-six years old, bilious temperament, was cured of a cyst on the right eyelid by Calc. c.$^{200}$, and in five weeks after the thirtieth. The small cyst disappeared perfectly in nine weeks.

